# Supplementary material for: Dual reporter genetic mouse models of pancreatic cancer identify an epithelial‐to‐mesenchymal transition‐independent metastasis program
Source: EMBO Mol Med. 2018 Aug 17;10(10):e9085. doi: 10.15252/emmm.201809085 (PMC6180301; doi:10.15252/emmm.201809085)

Appendix

Dual reporter genetic mouse models of pancreatic cancer identify an epithelial to mesenchymal transition independent metastasis program

Yang Chen[1], Valerie S. LeBleu[1], Julienne L. Carstens[1], Hikaru Sugimoto[1], Xiaofeng Zheng[1], Shruti Malasi[1], Dieter Saur[2-3] and Raghu Kalluri[1]

1 Department of Cancer Biology, Metastasis Research Center, University of Texas MD Anderson Cancer Center, Houston, TX 77054, USA
2 Department of Medicine II, Klinikum rechts der Isar, Technische Universität München, Ismaningerstrasse 22, 81675 München, Germany
3 German Cancer Research Center (DKFZ) and German Cancer Consortium (DKTK), Im Neuenheimer Feld 280, 69120 Heidelberg, Germany

Table of Contents:

1. Appendix Figure Legends
2. Appendix Figures

Appendix Figure Legends

Appendix Figure S1. Expression of Fsp1, Zeb1, and Vimentin in pancreatic cancer cells

(**A-C**) Representative images and quantification of percent double positive pancreatic cancers for YFP-lineage traced cancer cells from KPC;YFP (*LSL-Kras*$^{G12D/+}$*;Trp53*$^{R172H/+}$*;Pdx1-Cre;R26*$^{LSL-YFP}$) mice and Fsp1 (**A**), Zeb1 (**B**) or Vimentin (**C**). Reduction in EMT was observed in cancer cells from KPC;Twist1$^{loxP/loxP}$;YFP or KPC;Snai1$^{loxP/loxP}$;YFP mice harboring Twist or Snail conditional knockout (cKO). Percentages represent the present reduction of the cKO in comparison to the control mice. Panels below images are magnifications of areas in white boxes. N-values are distinct mice (5 visual fields were evaluated per mouse, n = 4 mice per group; results are presented as mean ± S.E.M.). Fsp1 staining (**A**): KPC versus KPC;Twist^{cKO} p = 0.0496, KPC versus KPC;Snai1^{cKO} p = 0.0259. Zeb1 staining (**B**): KPC versus KPC;Twist^{cKO} p = 0.0009, KPC versus KPC;Snai1^{cKO} p = 0.0006. Vimentin staining (**C**): KPC versus KPC;Twist^{cKO} p = 0.0444, KPC versus KPC;Snai1^{cKO} p = 0.0300. Significance determined by a One-way ANOVA, * $p < 0.05$, and *** $p < 0.001$. Scale bars, 20 μm.

Appendix Figure S2. Detection of αSMA expression in pancreatic cancer cells.

Representative images and quantification of primary PDAC tumors from KPC;YFP (LSL-Kras$^{G12D/+}$;Trp53$^{R172H/+}$;Pdx1-Cre;R26$^{LSL-YFP}$) mice examined for YFP and αSMA immunofluorescence staining (3 visual fields were evaluated per mouse, n = 4 mice; results are presented as mean ± S.E.M.). Arrows indicate YFP^{+}αSMA^{+} EMT cancer cells. Two distinct antibodies for αSMA (rabbit polyclonal antibody, Rb pAb; mouse monocolonal antibody, Mo mAb) were used. Arrow indicates YFP^{+}αSMA^{+} EMT cancer cell. Scale bars in all panels, 20 μm.

Appendix Figure S3. Examination of primary tumor and metastasis from KPF mice.

Representative images of PDAC and liver metastasis of KPF mice stained by H&E and immunohistochemistry (for CK19, αSMA, and Fsp1) methods. Scale bars, 100 μm. Some of the images shown here are also displayed in **Figure 1B-C**.

Appendix Figure S4. Examination of primary tumor and lung metastasis from KPF;αSMA-Cre;R26^{Dual} mice.

(**A**) Representative images of primary PDAC tumors from KPF;αSMA-Cre;R26^{Dual} mice examined for intrinsic EGFP and tdTomato signals, in combination with CK19 immunofluorescence co-staining. Red arrow indicates tdTomato^{+}CK19^{+} EMT cancer cell.

(**B**) Representative images of primary PDAC tumors from KPF;αSMA-Cre;R26^{Dual} mice. Circled areas indicate tdTomato^{+}CK19^{+} EMT cancer cell. Red arrows indicate tdTomato^{+}CK19^{-} αSMA-expressing myofibroblasts.
(**C**) Representative images of primary PDAC tumors from KPF;αSMA-Cre;R26^{Dual} mice examined for intrinsic EGFP and tdTomato signals, in combination with E-cadherin immunofluorescence co-staining. Scale bars in all panels, 20 μm.

Appendix Figure S5. Examination of tissues and isolated cancer cells from KPF;αSMA-Cre;R26^{Dual} mice.
(**A**) *Ex vivo* imaging of primary pancreatic tumor and lung of KPF;αSMA-Cre;R26^{Dual} mice visualized under either normal light or GFP excitation light (using a GFP band-pass filter 460-490 nm).
(**B**) KPF;αSMA-Cre;R26^{Dual} primary pancreatic cancer cells were treated with TGF-β (5 ng/mL, 96 h) for the *in vitro* induction of EMT and examined for intrinsic EGFP and tdTomato signals in combination with CK19 immunofluorescence co-staining. Scale bars, 20 μm.

Appendix Figure S6. EMT lineage tracing in KPF;αSMA-Cre;R26$^{mT/mG}$ mice.
(**A**) Genetic strategy to induce EGFP expression in *αSMA-Cre* lineage (either myofibroblasts or αSMA-expressing EMT cancer cells) in KPF;αSMA-Cre;R26$^{mT/mG}$ mice.
(**B**) Representative images of pre-malignant pancreatic tissues from KPF;αSMA-Cre;R26$^{mT/mG}$ mice examined for intrinsic tdTomato and EGFP signals, in combination with CK19 immunofluorescence staining. Scale bars, 20 μm. Scale bars in magnified panels, 10 μm.
(**C**) Representative images of metastases from KPF;αSMA-Cre;R26$^{mT/mG}$ mice examined for intrinsic tdTomato and EGFP signals, in combination with CK19 immunofluorescence staining. Scale bars, 50 μm. Scale bars in magnified panels, 10 μm.

Appendix Figure S7. EMT lineage tracing in KPF;Fsp1-Cre;R26^{Dual} mice.
(**A**) Schematic for EMT lineage tracing in KPF;Fsp1-Cre;R26^{Dual} mice.
(**B, C**) Representative images of co-localization (as indicated by arrows) between Fsp1-induced intrinsic tdTomato positivity and Vimentin (**B**) or Zeb1 (**C**) positivity by immunofluorescence staining in cancer cells of primary tumors from KPF;Fsp1-Cre;R26^{Dual} mice.
(**D**) Percentage of cells that are double-positive for both tdTomato and αSMA, Vimentin, or Zeb1 among all Fsp1-Cre/tdTomato^{+} cancer cells (3-4 visual fields were evaluated per mouse, n = 4 mice; results are presented as mean ± S.E.M.).
Scale bars in all panels, 20 μm. Scale bars in magnified panels, 10 μm.

Appendix Figure S8. The localization patterns of Fsp1 and αSMA among stromal fibroblasts in primary tumors of KPF;Fsp1-Cre;R26^{Dual} mice.

(**A**) Representative images of Fsp1-induced intrinsic tdTomato and αSMA immunofluorescence staining in stromal fibroblasts of primary tumors from KPF;Fsp1-Cre;R26^{Dual} mice.

(**B**) Representative images of Fsp1 and αSMA immunofluorescence stainings of primary tumors from KPF;Cre-negative;R26^{Dual} mice (which have EGFP expression in *Pdx-Flp* lineage cancer cells but no tdTomato expression).

(**C**) Representative images of Fsp1-induced intrinsic tdTomato and Fsp1 immunofluorescence staining of primary tumors from KPF;Fsp1-Cre;R26^{Dual} mice.

Scale bars in all panels, 20 μm.

Appendix Figure S1

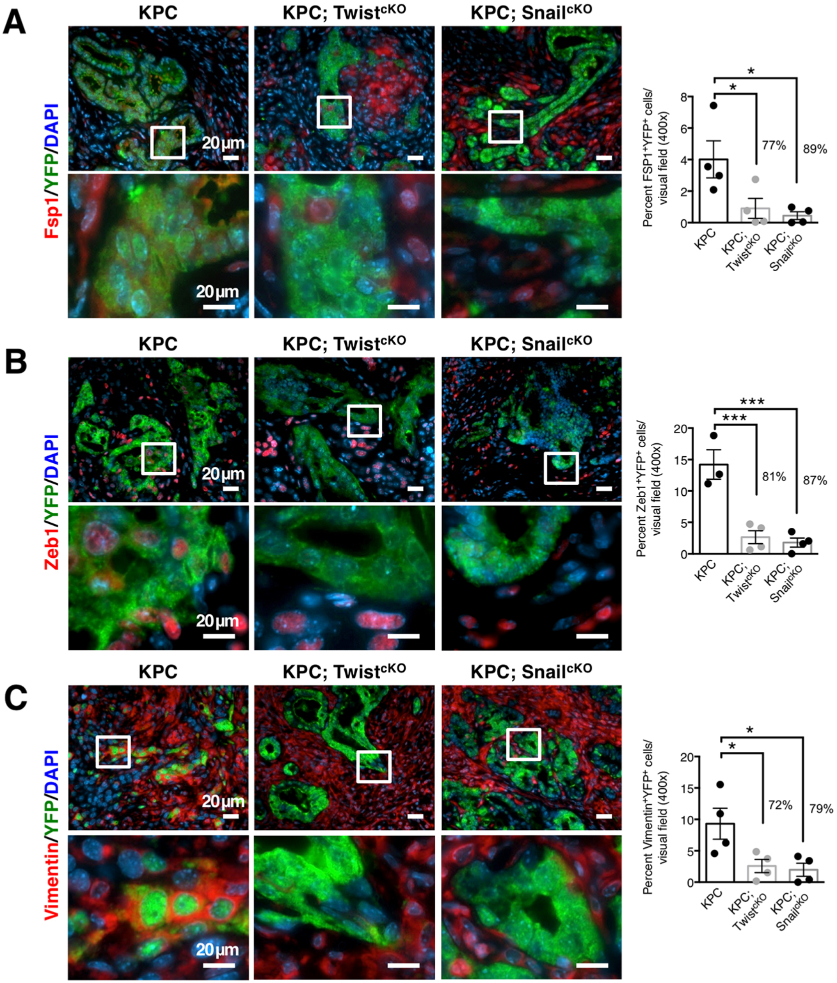

Appendix Figure S2

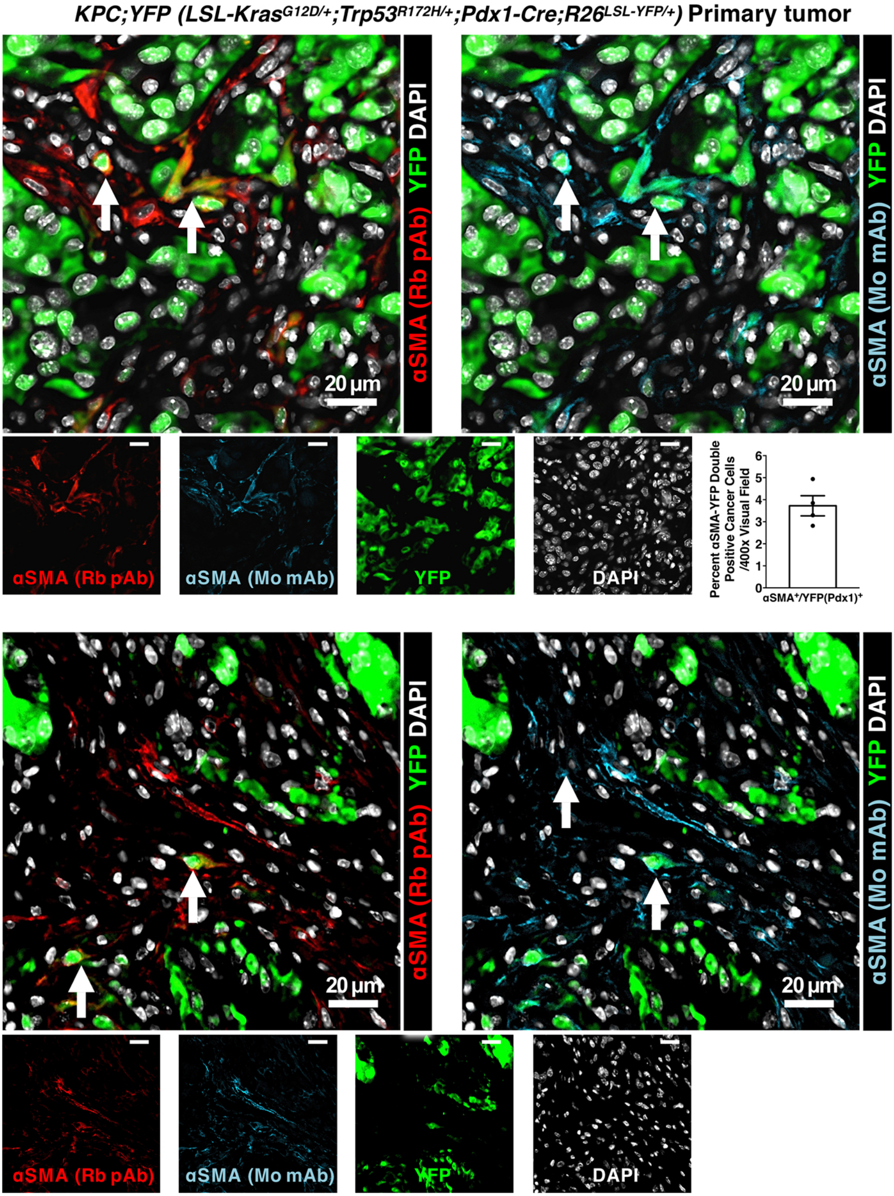

Appendix Figure S3

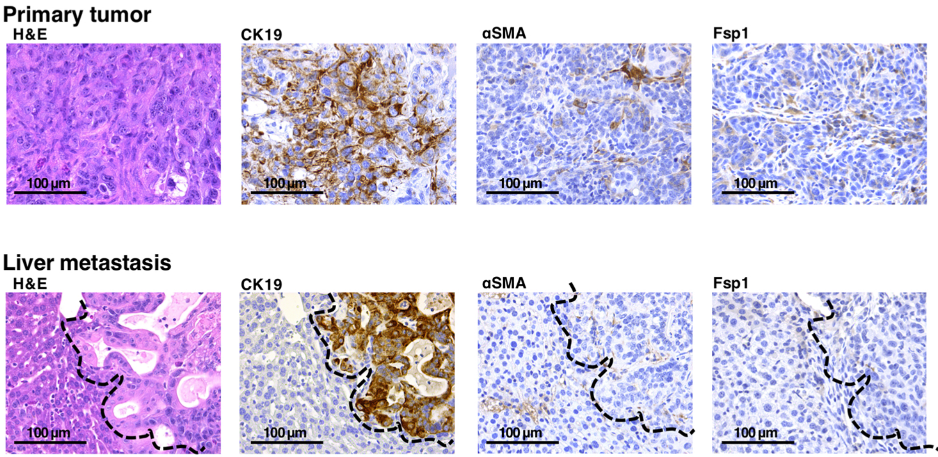

Appendix Figure S4

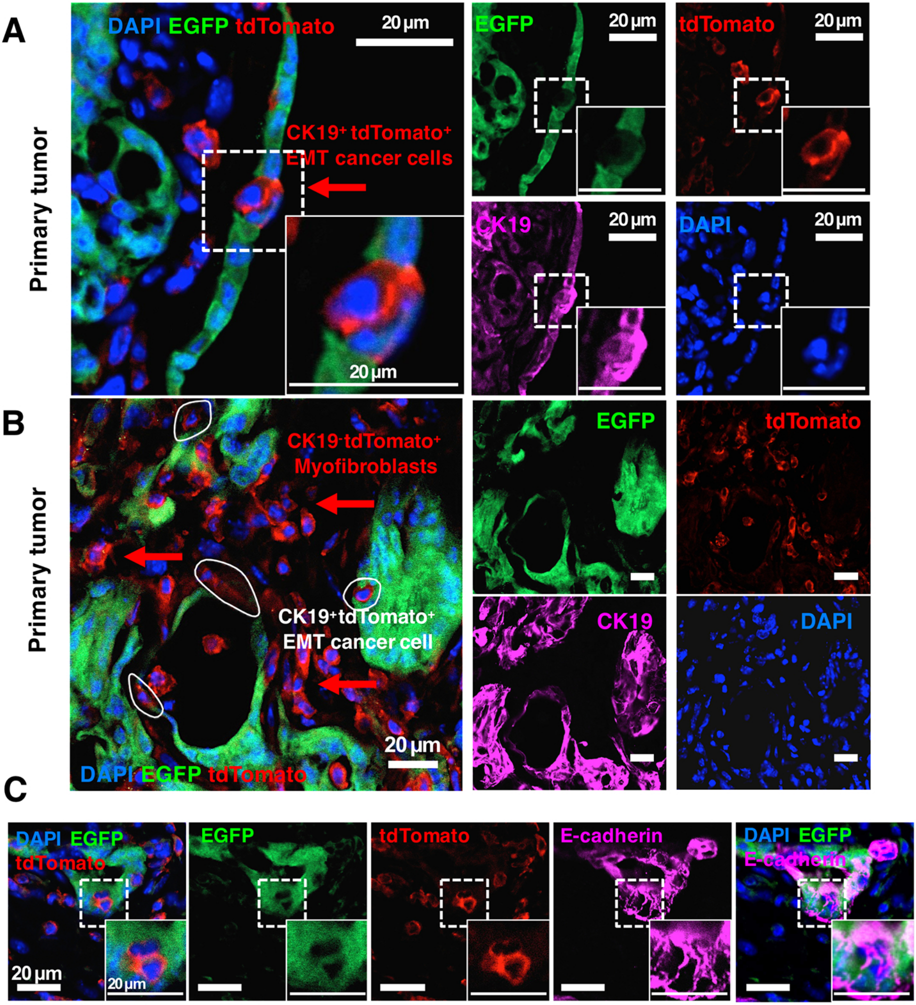

Appendix Figure S5

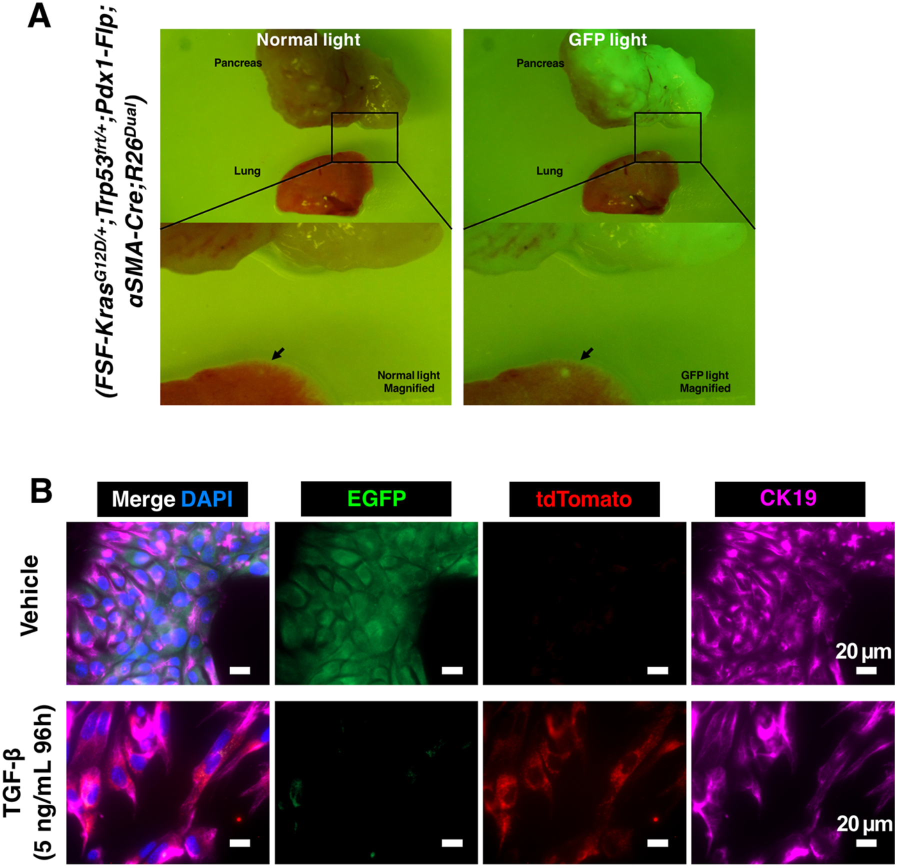

Appendix Figure S6

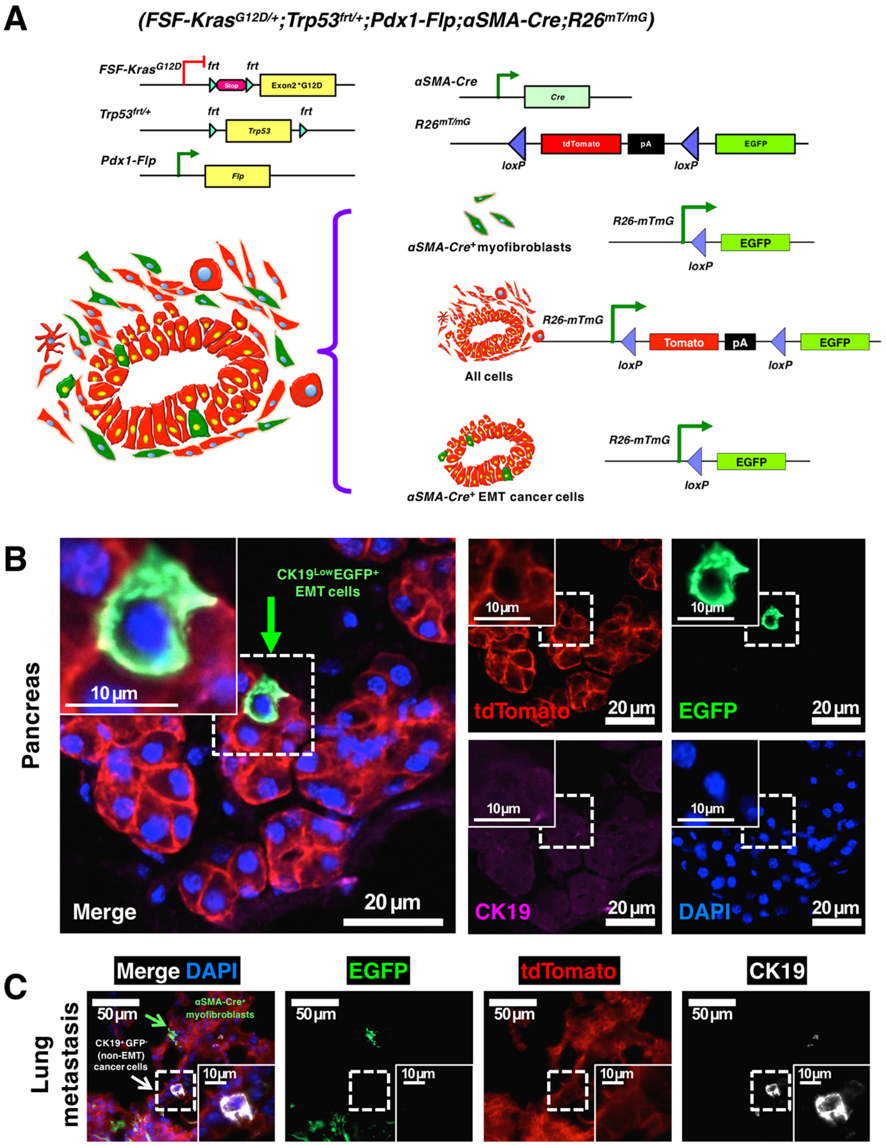

Appendix Figure S7

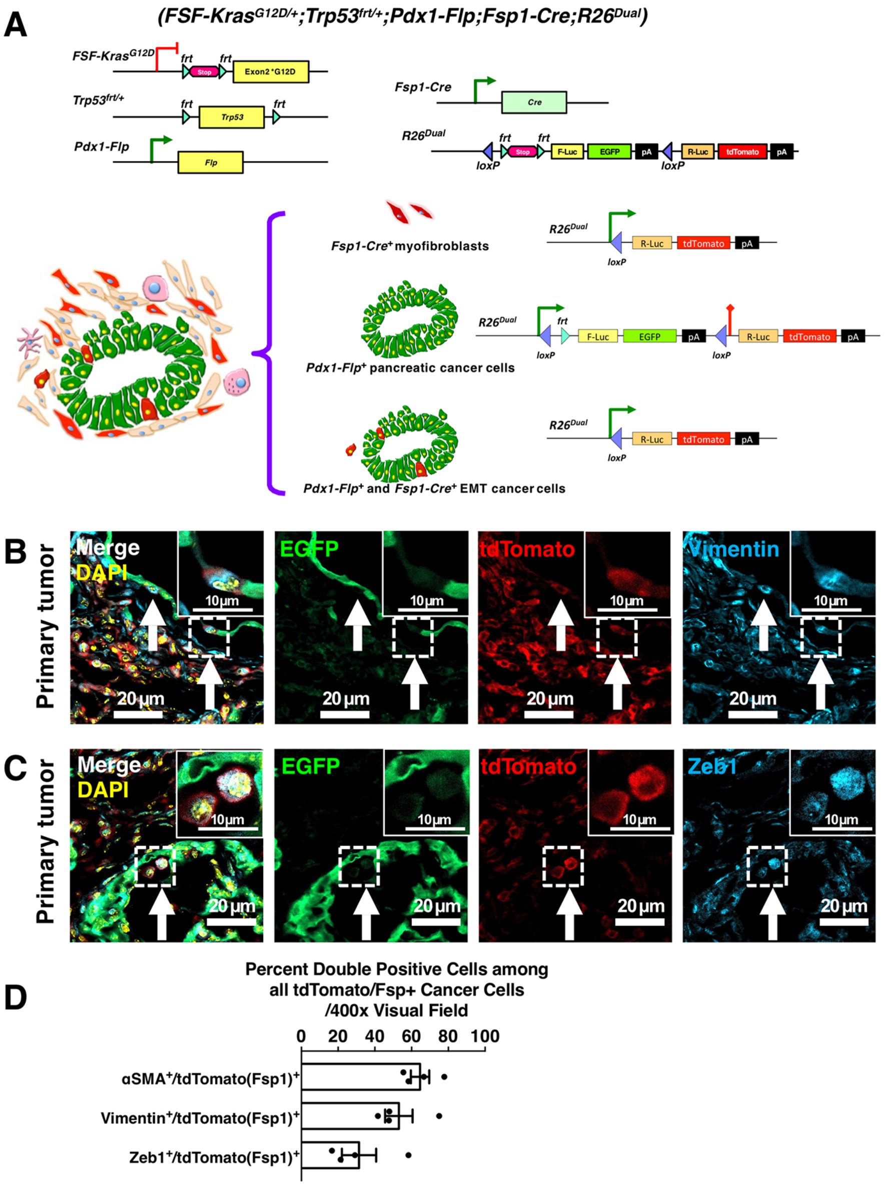

Appendix Figure S8

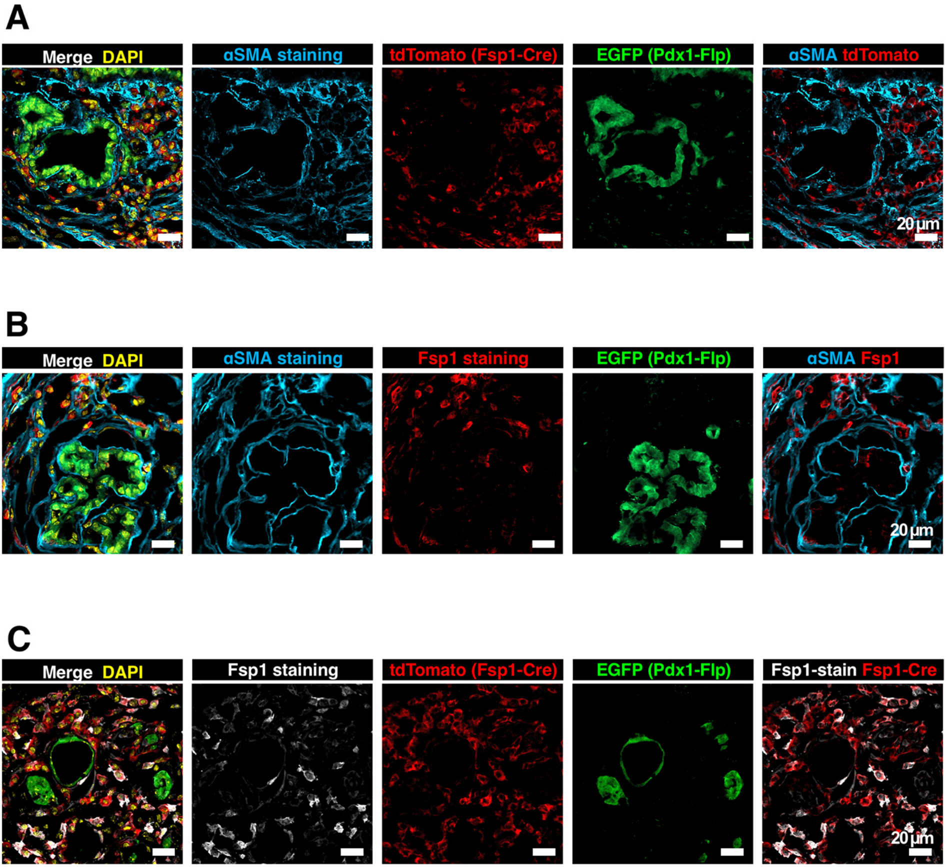

Supplement: Supplementary file 1 — Appendix [file EMMM-10-e9085-s001.pdf]
